# Supplementary material for: Abnormalities of the Halogen Bonds in the Complexes between Y2CTe (Y = H, F, CH3) and XF (X = F, Cl, Br, I)
Source: Molecules. 2022 Dec 3;27(23):8523. doi: 10.3390/molecules27238523 (PMC9739304; doi:10.3390/molecules27238523)
Supplement: Supplementary file 1 [file molecules-27-08523-s001.zip › molecules-2037720-supplementary.pdf]

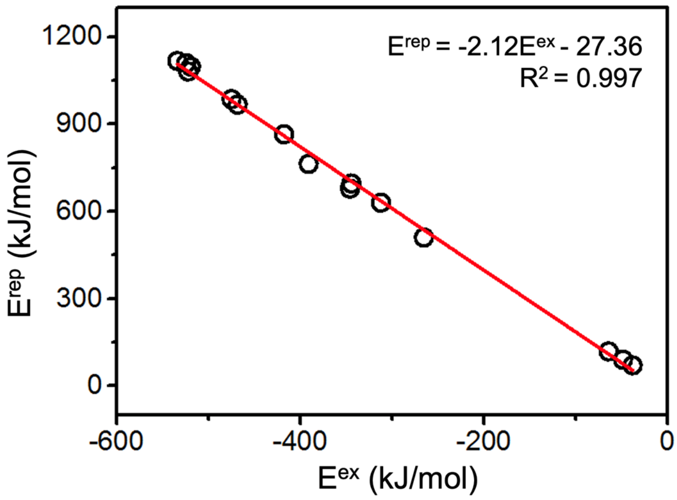


Figure S1. The relationship between repulsion energy $E^{rep}$ and exchange energy $E^{ex}$ in the complexes between $Y_2CTe$ (Y = H, F, and $CH_3$) and XF (X = H, F, Cl, Br, and I).

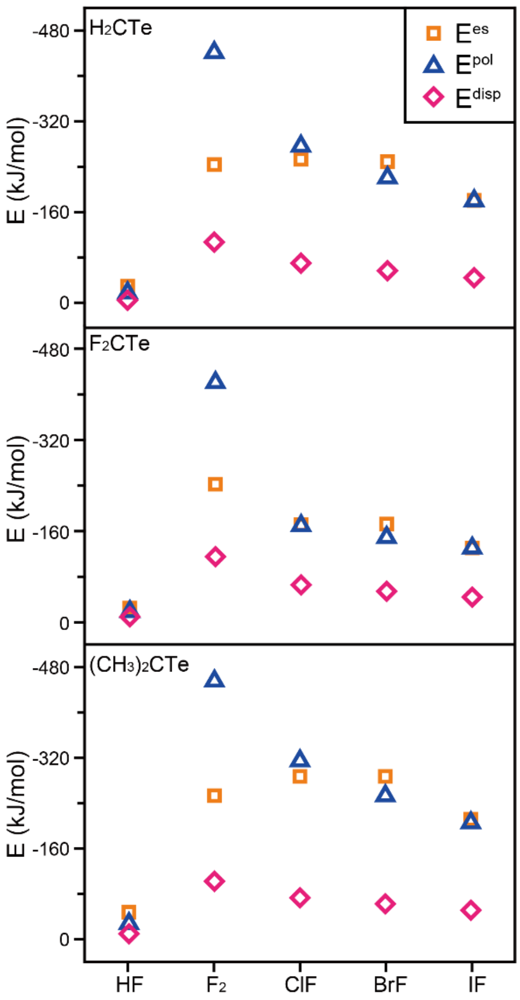


Figure S2. Electrostatic ($E^{es}$), polarization ($E^{pol}$), and dispersion ($E^{disp}$) energies in complexes between $Y_2CTe$ (Y = H, F, and $CH_3$) and XF (X = H, F, Cl, Br, and I).

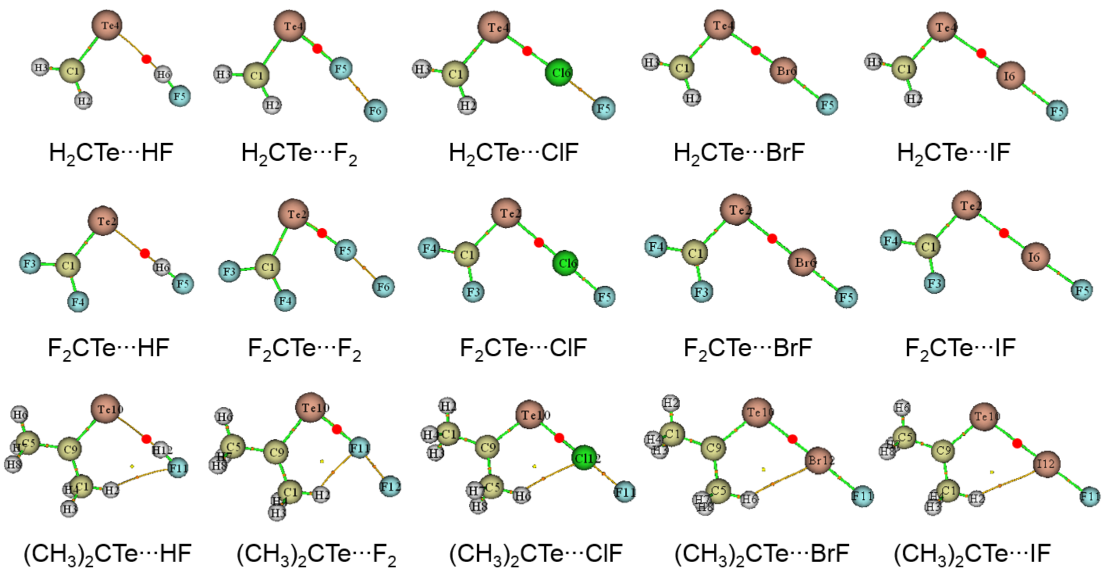


Figure S3. Molecular graphs of the complexes between $Y_2CTe$ (Y = H, F, $CH_3$) and XF (X = H, F, Cl, Br, I). Small red balls indicate the Te···X bond critical point.

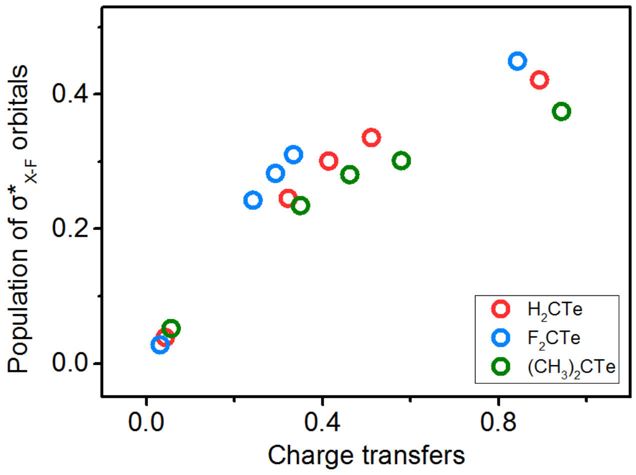


Figure S4. The relationship between the population of the $\sigma^*_{X\text{-}F}$ orbitals and the charge transfer in the complexes formed by $Y_2CTe$ (Y = H, F, and $CH_3$) and XF (X = H, F, Cl, Br, and I).

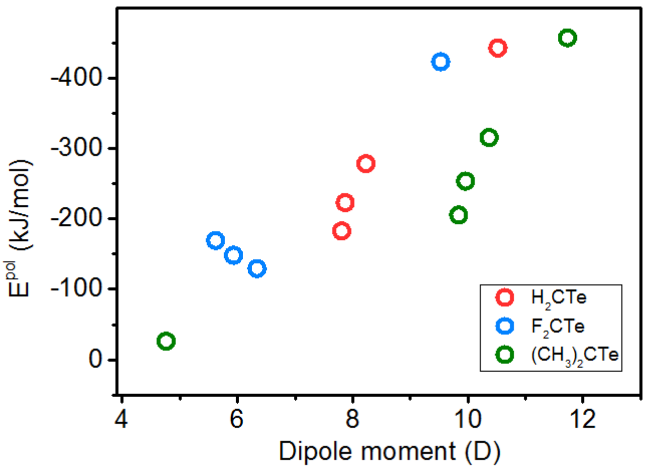


Figure S5. The relationship between the polarization energy $E^{pol}$ and the dipole moment of the complexes formed by $Y_2CTe$ (Y = H, F, and $CH_3$) and XF (X = F, Cl, Br, and I).

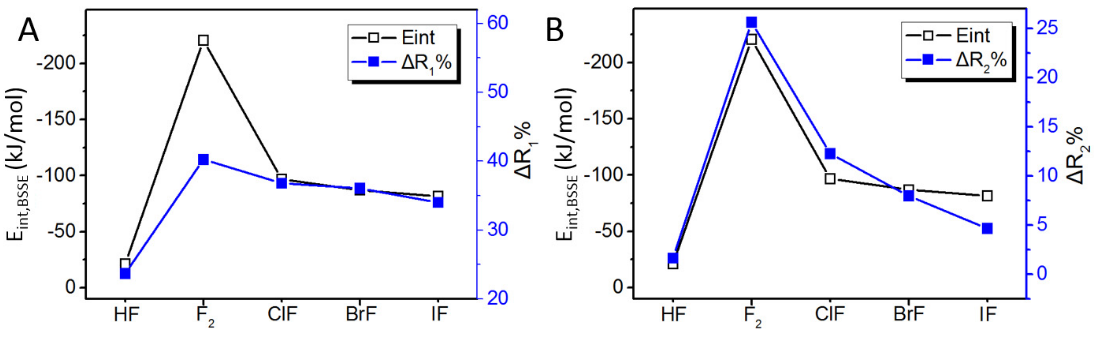


Figure S6. Trend comparison of $E_{int,BSSE}$ with $\Delta R_1\%$ (A) and $\Delta R_2\%$ (B) in $H_2CTe\cdots XF$ systems.

**The coordinates of optimized monomer $Y_2CTe$ (Y = H, F, $CH_3$), XF (X = H, F, Cl, Br, I), and their complexes.**

$H_2CTe$

| | | | |
|---|---|---|---|
| C | 0.00000000 | -1.66932500 | 0.00000000 |
| H | 0.92452000 | -2.23381100 | 0.00000000 |
| H | -0.92451100 | -2.23383600 | 0.00000000 |
| Te | 0.00000000 | 0.27853100 | 0.00000000 |

$F_2CTe$

| | | | |
|---|---|---|---|
| C | -0.00017100 | -1.14712200 | 0.00000000 |
| Te | 0.00000000 | 0.79898600 | 0.00000000 |
| F | -1.06263500 | -1.92596600 | 0.00000000 |
| F | 1.06274800 | -1.92564800 | 0.00000000 |

$(CH_3)_2CTe$

| | | | |
|---|---|---|---|
| C | 1.93856200 | -1.26029100 | -0.00000500 |
| H | 1.31811300 | -2.15179700 | 0.00023300 |
| H | 2.59352800 | -1.27798900 | -0.87673500 |
| H | 2.59369100 | -1.27772700 | 0.87664400 |
| C | 1.93856200 | 1.26029100 | -0.00000500 |
| H | 1.31811200 | 2.15179700 | 0.00018800 |
| H | 2.59365900 | 1.27774900 | 0.87666700 |
| H | 2.59356100 | 1.27796800 | -0.87671100 |
| C | 1.13007900 | 0.00000000 | -0.00004300 |
| Te | -0.82795900 | 0.00000000 | 0.00000100 |

HF

| | | | |
|---|---|---|---|
| F | 0.00000000 | 0.00000000 | 0.09218500 |
| H | 0.00000000 | 0.00000000 | -0.82966400 |

$F_2$

| | | | |
|---|---|---|---|
| F | 0.00000000 | 0.00000000 | 0.70058100 |
| F | 0.00000000 | 0.00000000 | -0.70058100 |

ClF

| | | | |
|---|---|---|---|
| F | 0.00000000 | 0.00000000 | -1.07141000 |
| Cl | 0.00000000 | 0.00000000 | 0.56721700 |

BrF

| | | | |
|---|---|---|---|
| F | 0.00000000 | 0.00000000 | -1.39848000 |
| Br | 0.00000000 | 0.00000000 | 0.35960900 |

IF

| | | | |
|---|---|---|---|
| F | 0.00000000 | 0.00000000 | -1.64106200 |
| I | 0.00000000 | 0.00000000 | 0.27867100 |

$H_2CTe\cdots HF$

| | | | |
|---|---|---|---|
| C | 0.40812600 | 1.67368900 | 0.00000100 |
| H | -0.54068900 | 2.19616900 | 0.00002900 |
| H | 1.31096600 | 2.27166100 | -0.00002800 |
| Te | 0.48693200 | -0.27130600 | 0.00000000 |
| F | -2.94540300 | -0.01992700 | -0.00000400 |
| H | -2.03088400 | -0.22270500 | 0.00002600 |

$H_2CTe\cdots F_2$

| | | | |
|---|---|---|---|
| C | 0.51970200 | 1.67251800 | 0.00002200 |
| H | -0.48672200 | 2.07748600 | 0.00022600 |
| H | 1.37653600 | 2.33277200 | -0.00015500 |
| Te | 0.71822400 | -0.24278900 | -0.00001800 |
| F | -1.43262200 | -0.26363900 | 0.00020500 |
| F | -3.16245400 | 0.06137700 | -0.00012500 |

$H_2CTe\cdots ClF$

| | | | |
|---|---|---|---|
| C | -1.25498700 | 1.62469400 | -0.00006500 |
| H | -0.41452400 | 2.31006100 | -0.00044800 |
| H | -2.26293200 | 2.01928900 | 0.00060000 |
| Te | -0.91890200 | -0.28308800 | 0.00005700 |
| F | 3.42150000 | 0.19550700 | 0.00047500 |
| Cl | 1.59981200 | -0.06567700 | -0.00041200 |

$H_2CTe\cdots BrF$

| | | | |
|---|---|---|---|
| C | 1.65383900 | 1.61692300 | 0.00009200 |
| H | 0.83845500 | 2.33141600 | -0.00054900 |
| H | 2.67380200 | 1.97959400 | 0.00011700 |
| Te | 1.26240200 | -0.28281600 | 0.00000400 |
| F | -3.29294600 | 0.22141800 | 0.00008800 |
| Br | -1.41267700 | -0.03711000 | -0.00003200 |

$H_2CTe\cdots IF$

| | | | |
|---|---|---|---|
| C | 2.01881700 | 1.61089600 | -0.00004800 |
| H | 1.22353500 | 2.34720300 | 0.00017000 |
| H | 3.04781300 | 1.94746100 | 0.00009500 |
| Te | 1.58697200 | -0.28253200 | -0.00001900 |
| F | -3.29898500 | 0.22575100 | -0.00016700 |
| I | -1.30596000 | -0.02453100 | 0.00004800 |

$F_2CTe\cdots HF$

| | | | |
|---|---|---|---|
| C | 1.08366000 | 0.73214800 | -0.00000800 |
| Te | -0.12459800 | -0.79958200 | -0.00006800 |
| F | 2.39464000 | 0.67109600 | 0.00066800 |
| F | 0.73141600 | 1.99450700 | -0.00066100 |
| F | -2.88903700 | 1.37711700 | 0.00043200 |
| H | -2.15602300 | 0.80089100 | -0.00037400 |

$F_2CTe\cdots F_2$

| | | | |
|---|---|---|---|
| C | 0.68580800 | 1.14353100 | 0.00010600 |
| Te | 0.47860400 | -0.80989200 | 0.00001200 |
| F | 1.84938300 | 1.73902900 | -0.00019500 |
| F | -0.26276700 | 2.01553500 | 0.00017100 |
| F | -1.59342000 | -0.27577200 | -0.00002600 |
| F | -3.21566600 | 0.43822900 | -0.00008800 |

$F_2CTe\cdots ClF$

| | | | |
|---|---|---|---|
| C | 1.33414700 | 0.95989900 | -0.00001000 |
| Te | 0.53636300 | -0.82911500 | -0.00001000 |
| F | 0.68265900 | 2.08546600 | -0.00023500 |
| F | 2.62071700 | 1.20135800 | 0.00025300 |
| F | -3.61192200 | 0.70552900 | 0.00022400 |
| Cl | -1.94816700 | 0.08372900 | -0.00009400 |

$F_2CTe\cdots BrF$

| | | | |
|---|---|---|---|
| C | -1.68704400 | 0.96627900 | 0.00062800 |
| Te | -0.89839900 | -0.82677600 | -0.00008400 |
| F | -1.03167800 | 2.09068700 | -0.00066500 |
| F | -2.97261900 | 1.21346600 | 0.00040700 |
| F | 3.51394400 | 0.62883100 | 0.00013200 |
| Br | 1.75006300 | 0.05136700 | 0.00004900 |

$F_2CTe\cdots IF$

| | | | |
|---|---|---|---|
| C | -2.02087300 | 0.97456600 | 0.00010700 |
| Te | -1.23738400 | -0.82094400 | 0.00007200 |
| F | -1.36556400 | 2.09979500 | -0.00269300 |
| F | -3.30660300 | 1.22305500 | 0.00298500 |
| F | 3.54311400 | 0.57406800 | 0.00425600 |
| I | 1.63454100 | 0.03338500 | -0.00085500 |

$(CH_3)_2CTe\cdots HF$

| | | | |
|---|---|---|---|
| C | -0.68363000 | 2.12169900 | -0.00005900 |
| H | 0.39320300 | 2.26186800 | -0.00050600 |
| H | -1.11097000 | 2.62003100 | -0.87580900 |
| H | -1.11015900 | 2.61976100 | 0.87623500 |

| | | | |
|---|---|---|---|
| C | -2.57337200 | 0.45359000 | 0.00006300 |
| H | -2.83391000 | -0.60092200 | 0.00033700 |
| H | -3.01824200 | 0.93473100 | 0.87639400 |
| H | -3.01825300 | 0.93426600 | -0.87651200 |
| C | -1.09553900 | 0.68486600 | -0.00003400 |
| Te | 0.18246200 | -0.80007500 | -0.00000600 |
| F | 2.79696000 | 1.39584500 | 0.00005700 |
| H | 2.15293100 | 0.71064800 | -0.00015000 |

$(CH_3)_2CTe\cdots F_2$

| | | | |
|---|---|---|---|
| C | 0.00709700 | 2.15778100 | 0.00031500 |
| H | 1.05438900 | 1.86921100 | -0.00309000 |
| H | -0.21188300 | 2.78120700 | -0.87251500 |
| H | -0.20652400 | 2.77505300 | 0.87897900 |
| C | -2.38518400 | 1.32179000 | -0.00038300 |
| H | -3.02254400 | 0.44135600 | -0.00132800 |
| H | -2.61746300 | 1.93179600 | 0.87718700 |
| H | -2.61697700 | 1.93361200 | -0.87670200 |
| C | -0.92595300 | 1.00005900 | -0.00030000 |
| Te | -0.32703800 | -0.85460500 | 0.00002400 |
| F | 1.72569500 | -0.13125500 | 0.00030200 |
| F | 3.21332900 | 0.77897000 | -0.00047800 |

$(CH_3)_2CTe\cdots ClF$

| | | | |
|---|---|---|---|
| C | 2.92247500 | 0.87353200 | 0.00008200 |
| H | 3.35275400 | -0.12518200 | -0.00016400 |
| H | 3.28370800 | 1.41839500 | -0.87681900 |
| H | 3.28353400 | 1.41784300 | 0.87740700 |
| C | 0.76078800 | 2.19989800 | -0.00007300 |
| H | -0.32356400 | 2.13202900 | -0.00007100 |
| H | 1.09027500 | 2.76548500 | 0.87692300 |
| H | 1.09032400 | 2.76548200 | -0.87703600 |
| C | 1.42889600 | 0.87080800 | -0.00003500 |
| Te | 0.43868800 | -0.81687500 | -0.00001200 |
| F | -3.63261700 | 0.79339600 | 0.00003800 |
| Cl | -1.91577700 | 0.07632000 | 0.00001200 |

$(CH_3)_2CTe\cdots BrF$

| | | | |
|---|---|---|---|
| C | 3.27124700 | 0.89441800 | -0.00000500 |
| H | 3.70841000 | -0.10122500 | -0.00038200 |
| H | 3.62881000 | 1.44210900 | -0.87672900 |
| H | 3.62870800 | 1.44131200 | 0.87728200 |
| C | 1.09925100 | 2.20323500 | -0.00000800 |
| H | 0.01569400 | 2.12093400 | -0.00026900 |

| | | | |
|---|---|---|---|
| H | 1.42104200 | 2.77266600 | 0.87736100 |
| H | 1.42153400 | 2.77285300 | -0.87707900 |
| C | 1.77777000 | 0.87948800 | -0.00001600 |
| Te | 0.79941100 | -0.81666500 | -0.00000800 |
| F | -3.53051000 | 0.70451400 | -0.00005700 |
| Br | -1.72881700 | 0.05184100 | 0.00002600 |

$(CH_3)_2CTe\cdots IF$

| | | | |
|---|---|---|---|
| C | 1.44947400 | 2.21367000 | 0.00006600 |
| H | 0.36497200 | 2.13948200 | 0.00072500 |
| H | 1.77466200 | 2.78222700 | 0.87686300 |
| H | 1.77344700 | 2.78163300 | -0.87757300 |
| C | 3.61290500 | 0.89578700 | -0.00005500 |
| H | 4.04605500 | -0.10147400 | 0.00054100 |
| H | 3.97255200 | 1.44120800 | -0.87746900 |
| H | 3.97278300 | 1.44248900 | 0.87644400 |
| C | 2.11895600 | 0.88530300 | 0.00005000 |
| Te | 1.13887600 | -0.81134900 | 0.00001800 |
| F | -3.55773600 | 0.63235600 | 0.00004500 |
| I | -1.62631000 | 0.03858100 | -0.00002300 |
